# Brucellosis associated with acute kidney injury and pancytopenia: a case report and review of the literature

**DOI:** 10.3389/fmed.2026.1780291

**Published:** 2026-06-12

**Authors:** Ya Wang, Fangqiu Yu, Wei Wang

**Affiliations:** 1 Department of Nephrology, The Second Hospital and Clinical Medical School, Lanzhou University, Lanzhou, Gansu, China; 2 Department of Urology, The Second Hospital and Clinical Medical School, Lanzhou University, Lanzhou, Gansu, China; 3 Department of Urology, Shenzhen Hospital, Southern Medical University, Shenzhen, China

**Keywords:** acute kidney injury, brucellosis, fever, haemophagocytic syndrome, pancytopenia

## Abstract

Brucella infection frequently involves multiple organ systems, such as muscles, bones, liver, spleen, gastrointestinal tract, and others. Its clinical manifestations lack specificity. Acute kidney injury complicated by haemophagocytic syndrome is particularly rare in the course of brucellosis. We described a 51-year-old man with brucellosis presenting with acute kidney injury and haemophagocytic syndrome. His primary manifestations included unexplained fever, progressive pancytopenia, progressive elevation of serum creatinine, imaging demonstrating increased renal cortical echogenicity, and positive blood culture results for Brucella. Following combined anti-Brucella therapy and etoposide (VP-16) chemotherapy, the patient's symptoms and renal function improved significantly. This case highlights the need to consider rare pathogens in cases of unexplained fever, as the clinical features of haemophagocytic syndrome lack specificity. Detailed history-taking, definitive laboratory findings, and awareness of rare complications of brucellosis facilitate early diagnosis and management.

## Introduction

Brucellosis is an infectious disease caused by Brucella bacteria, ranking among the world's most prevalent zoonotic infections (1, 2). China reports approximately 40,000 new cases annually, predominantly distributed across the Northeast, Northwest, and Northern regions, constituting a significant public health concern (3). Human infection routes include consumption of undercooked meat, unpasteurized dairy products, or close contact with animals and their excretions. Consequently, the disease exhibits strong occupational relevance, predominantly affecting meat processing personnel, slaughterhouse workers, veterinarians, and other groups directly handling animals and animal products (4). Brucella infection can affect multiple organs, including the musculoskeletal system, liver and spleen, gastrointestinal tract, nervous system, and urogenital system. Renal involvement is less common and may present as interstitial nephritis, pyelonephritis, IgA nephropathy, focal segmental glomerulosclerosis, or mixed cryoglobulinemia, among other forms (5, 6). Clinical manifestations are often atypical, with common signs of infection including fever, fatigue, night sweats, myalgia, arthralgia, malaise, and hepatosplenomegaly or lymphadenopathy (7). Symptoms such as osteoarticular inflammation, orchitis, hepatitis, pneumonia, and neurological disorders, may arise in any body system following infection (8, 9). Owing to atypical clinical presentations, diagnostic challenges, and inadequate surveillance, numerous cases remain undiagnosed, rendering currently reported figures a conservative estimate (10). Therefore we report a case of a 51-year-old man who developed acute kidney injury and haemophagocytic syndrome secondary to brucellosis. Although rare and rapidly progressive, the patient fortunately achieved a favorable prognosis following antimicrobial therapy.

## Case report

A 51-year-old man was admitted, presenting with “fever accompanied by elevated serum creatinine for 2 weeks”. The fever developed 2 weeks prior to admission and was accompanied by chills, with a peak temperature of 40 C. The patient has not experienced coughing or sputum production, nor any other symptoms such as abdominal pain or diarrhea. Investigations at a local hospital revealed the following: Urinalysis revealed protein 3+, elevated red blood cells and white blood cells, along with a 24-h urine protein quantification of 12.24 g/24 h (normal range: < 0.15 g/24 h). Blood count showed: WBC 1.2 × 10^9^/L (normal range: 4–10), HGB 133 g/L (normal range: 130–175), PLT 33 × 10^9^/L (normal range: 100–300). Blood biochemistry showed: BUN 11.8 mmol/L (normal range: 2.5–7.9), CREA 161 μmol/L (normal range: 53–106), UA 403 μmol/L (normal range: 150–420), ALB 26 g/L (normal range: 40–55), GLO 43 g/L (normal range: 20–40), Ca 2.05 mmol/L (normal range: 2.15–2.55), complement C3 0.28 g/L (normal range: 0.9–1.8). ANA and autoantibodies were negative. Serum protein electrophoresis demonstrates a positive IgG-κ band. Bone marrow aspiration revealed 2% plasma cells. Chest CT showed no abnormalities. Abdominal CT demonstrated heterogeneous hepatic density reduction and splenomegaly. Liver transient elastography indicated significant fibrosis. The local hospital administered meropenem and moxifloxacin for infection control. Persistent fever, progressive elevation of serum creatinine, and a complete blood count showing progressive pancytopenia led to admission to our hospital. The patient has a history of hepatitis B and hypertension. According to our medical records, the patient has no history of medication use associated with complications.

**Figure 1:**
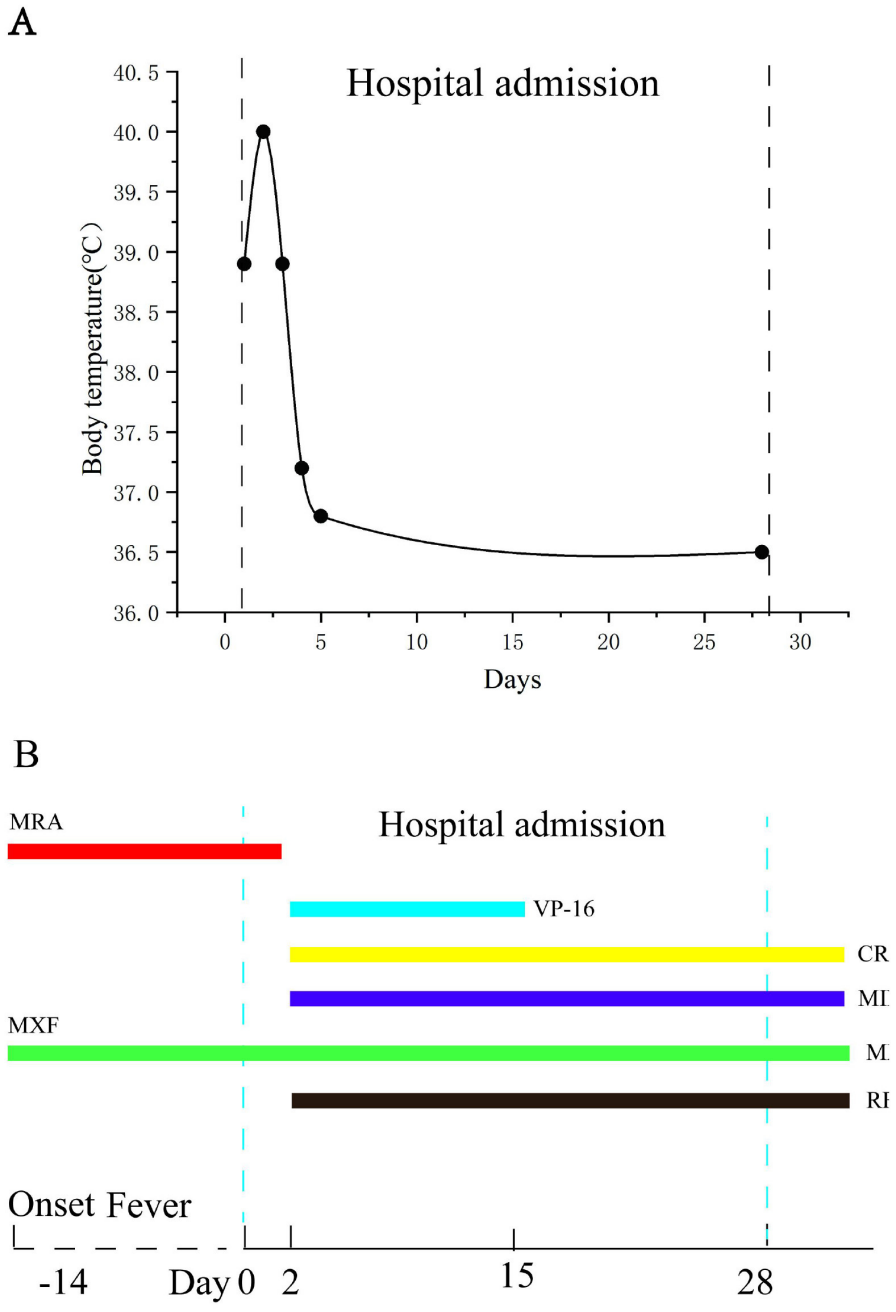
The patient's clinical presentation and treatment timeline. **(A)** Timeline of the patient's body temperature. **(B)** Timeline of the patient's treatment. MRA, meropenem; MXF, moxifloxacin; VP-16, etoposide; CRO, ceftriaxone; MINO, minocycline; RFP, rifampicin.

On admission, physical examination findings of patient were as follows: Temperature 39.8°C (Figure 1), pulse rate 102 beats per minute, respiratory rate 20 breaths per minute, and blood pressure 150/90 mmHg. There was no palpable enlargement of superficial lymph nodes, no eyelid oedema, no jaundice of the skin, and the conjunctivae were pale. No abnormal findings were noted on examination of the heart, lungs, or abdomen, and there was no oedema of the lower limbs.

Supplementary test results are as follows: Blood count: WBC 1.1 × 10^9^/L (normal range: 3.5–9.5), HGB 100 g/L (normal range: 130–175), PLT 26 × 10^9^/L (normal range: 100–300); blood biochemistry: BUN 9.4 mmol/L (normal range: 2.5–7.9), CREA 217 μmol/L (normal range: 53–106; Figure 2), UA 681 μmol/L (normal range: 150–420), CO_2_ 11.9 mmol/L (normal range: 23–31), ALB 19.2 g/L (normal range: 40–55), ALT 105 U/L (normal range: 9–50), AST 82 U/L (normal range: 15–40), TG 2.1 mmol/L (normal range: 0.56–1.70), PCT 0.98 ng/mL (normal range: < 0.3), and ferritin >2000 ng/mL (normal range: 15–300). The respiratory panel for nine viral markers was negative. EBV testing revealed a positive EBV antigen IgG antibody, a negative G+GM test, a negative tuberculin skin test (TSRPT), and a negative ASO test. Serum free light chains κ/λ were normal. Immunofixation electrophoresis showed IgG-κ type M protein positivity. Complement C3 and C4 levels were decreased. The tiger red tube agglutination test was positive, This indicates the possible presence of Brucella antibodies in the patient's body. Blood culture revealed Brucella. However, blood culture alone cannot directly identify the species of Brucella; it merely isolates live bacteria from the blood. Only MALDI-TOF MS and PCR can identify the specific species. Urinalysis showed protein 3+, with 24-h urine protein quantification at 12 g/24 h (normal range: < 0.15). Urinary tract color Doppler ultrasound findings: The left kidney measures 11.5 × 6.8 × 5.5 cm, with a cortical thickness of 0.87 cm; the right kidney measures 12.1 × 6.5 × 5.7 cm, with a cortical thickness of 0.87 cm and cortical echogenicity enhancement. No abnormalities were noted in the ureters or bladder. Cardiac ultrasound findings: The interventricular septum is thickened to 12 mm, with left ventricular enlargement and an ejection fraction of 60%. Subcutaneous fat biopsy from the abdomen showed negative Congo red staining. Bone marrow aspiration results: Tripartite hyperplasia of the bone marrow suggesting infection, 3% plasma cells, and haemophagocytosis observed. sCD25 was 4,872 U/mL (normal range: 200–1000), and NK cell activity was 2.6%.

**Figure 2:**
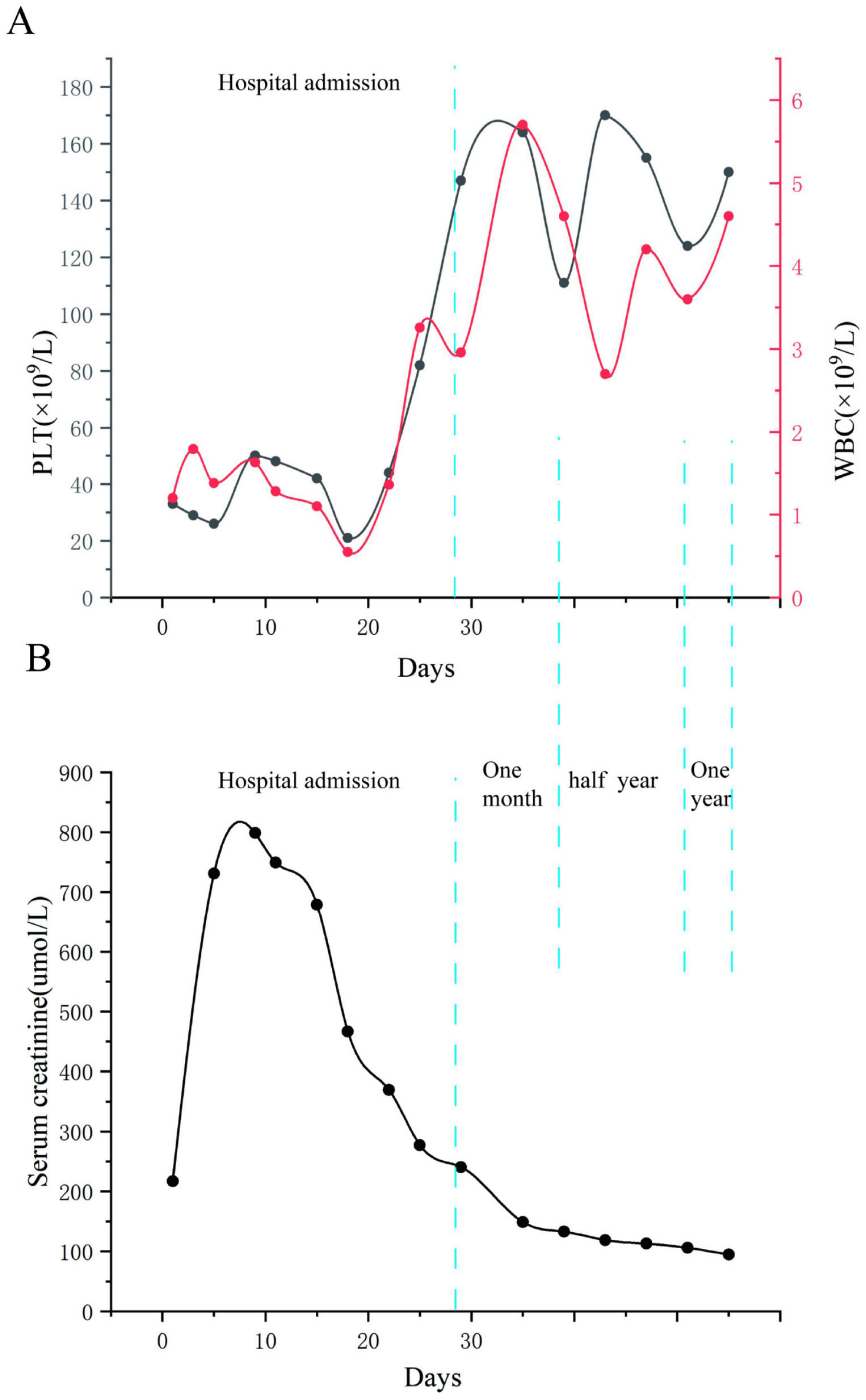
Changes in the patient's clinical indicators. **(A)** Changes in the patient's white blood cell (WBC) and platelet counts (PLT). **(B)** Creatinine changes over the treatment timeline.

Post-admission management: Following admission, the patient developed a persistent fever with a peak temperature of 40°C. Meropenem was administered to control the infection, yet the fever persisted. On the third day post-admission, the patient presented with oedema, oliguria, and an elevated serum creatinine level of 730.9 μmol/L, alongside a decrease in platelet count to 26 × 10^9^/L. Immediate central venous catheterisation was performed to initiate continuous renal replacement therapy (CRRT). Following a multidisciplinary consultation, the diagnosis was established as brucellosis complicated by acute kidney injury and haemophagocytic syndrome. Quadruple anti-brucellosis therapy was administered: ceftriaxone 2 g intravenous infusion q12h, minocycline 0.1 g orally q12h, moxifloxacin 0.4 g intravenous infusion q12h, and rifampicin 250 mg intravenous infusion q12h (Figure 1). For haemophagocytic syndrome, chemotherapy with VP-16 combined with dexamethasone was administered twice weekly for the first 2 weeks. Concurrent supportive therapies included CRRT, antiviral treatment for hepatitis B, hepatoprotective agents, and nutritional support. The following day after treatment for brucellosis, the patient's temperature returned to the normal range. Blood tests indicated a gradual increase in all three blood cell lines, with improved liver and kidney function compared to previous readings. Following the third chemotherapy cycle, the patient experienced recurrent hematological decline, attributed to etoposide-induced myelosuppression. Treatment of VP-16 was therefore discontinued for observation. Subsequently, urine output gradually normalized. Follow-up blood tests revealed: WBC 4.2 × 10^9^/L, HGB 118 g/L, PLT 137 × 10^9^/L, ferritin 800 ng/mL, triglycerides within the normal range, complement levels restored to normal, and serum immunofixation electrophoresis negative. Following discontinuation of dialysis, serum creatinine decreased to 270 μmol/L. The dialysis catheter was subsequently removed, dialysis was ceased, and the patient was discharged in improved condition. Dexamethasone was tapered and discontinued according to the HLH-2004 protocol. The patient was advised to continue oral quadruple anti-brucellosis medication and attend regular follow-up appointments at the Infectious Diseases, Hematology, and Nephrology outpatient clinics.

Antiviral prophylaxis for hepatitis B reactivation was initiated concurrently with HLH-directed chemotherapy. The patient was HBsAg-positive with baseline undetectable HBV DNA; however, given the planned administration of etoposide plus high-dose dexamethasone (per HLH-2004 protocol), he was at high risk for HBV reactivation. Entecavir 0.5 mg orally once daily was commenced on the same day as VP-16 initiation and continued throughout the chemotherapy course. This strategy aligns with current international guidelines (EASL, AASLD, and ASCO) recommending prophylaxis with high-barrier nucleostide analogs (entecavir or tenofovir) in all HBsAg-positive patients receiving chemotherapy or immunosuppressive therapy, irrespective of baseline HBV DNA levels (11). Prophylaxis was maintained for 12 months following completion of dexamethasone, with regular monitoring of ALT and HBV DNA every 4–8 weeks. No hepatic flare or virological breakthrough was observed during follow-up.

Follow-up: 1 week post-discharge, a routine urine test showed negative proteinuria. Blood count revealed: WBC 5.8 × 10^9^/L, HGB 92 g/L, PLT 278 × 10^9^/L. Blood biochemistry indicated: CREA 218 μmol/L, ALT 16 U/L, AST 17 U/L, TG 1.62 mmol/L, ALB 44 g/L. One month post-discharge: urinalysis showed negative proteinuria. Blood count: WBC 5.37 × 10^9^/L, HGB 112 g/L, PLT 116 × 10^9^/L, CREA 119 μmol/L. Six months post-discharge, follow-up urine analysis showed negative proteinuria. Blood tests indicated: WBC 3.2 × 10^9^/L, HGB 143 g/L, PLT 124 × 10^9^/L, CREA 106 μmol/L, ALB 44 g/L, with normal liver enzyme levels (Table 1). One year after discharge, the patient reported complete restoration of his premorbid functional status, with unrestricted ability to perform activities of daily living and resume regular work. He described his quality of life as “fully recovered” with no residual fatigue, musculoskeletal limitations, or dietary restrictions. This favorable functional outcome, underscores the importance of early recognition and aggressive multimodal therapy in brucellosis complicated by acute kidney injury and hemophagocytic syndrome.

**Table 1:** Comparison of clinical indicators during the patient's treatment process disease.

Projection	Day1	Day4	Day28	1 week after discharge	1 month after discharge	Half year after discharge
WBC(× 10^9^/L)	1.1	1.38	2.96	5.8	5.37	3.2
HGB(g/L)	100	101	118	92	112	143
PLT(× 10^9^/L)	26	26	147	278	116	124
CREA(μmol/L)	217.2	730.9	240.7	218	119	106
Proteinuria	+	+	–	–	–	–
Temperature (°C)	39.8	37.2	normal	normal	Normal	normal

## Discussion

This patient was a middle-aged man presenting with acute onset. Renal manifestations included significant proteinuria and impaired renal function; extrarenal manifestations comprise fever, progressive pancytopenia, decreased complement levels, splenomegaly, and monoclonal immunoglobulin disease. The patient had a history of hepatitis B and hypertension. The differential diagnosis should include: (1) Systemic lupus erythematosus: This condition predominantly affects women of childbearing age. Renal manifestations may include haematuria and proteinuria, with or without renal impairment. Extrarenal symptoms may present as fever, musculoskeletal pain, cytopenia, and serous effusions. It is characterized by decreased complement levels and is typically positive for immunological markers such as antinuclear antibodies, dsDNA antibodies, and Sm antibodies. This patient tested negative for autoantibodies on two occasions, thus ruling out systemic lupus erythematosus. (2) Hepatitis B-related cirrhosis: This condition may present with splenomegaly, hypersplenism, and cytopenia, potentially leading to secondary renal impairment. The patient has a history of hepatitis B. Comprehensive investigations indicate increased liver stiffness, though not yet meeting the diagnostic criteria for cirrhosis. Furthermore, cirrhosis cannot account for extrarenal manifestations such as fever and low complement levels, thus ruling out this diagnosis. (3) Cryoglobulinaemia: Typically associated with monoclonal immunoglobulins, it may arise secondary to viral hepatitis or autoimmune diseases. Renal manifestations include haematuria, proteinuria, or renal insufficiency, while extrarenal symptoms encompass cutaneous purpura, Raynaud's phenomenon, arthralgia, peripheral neuropathy, and fever. This patient tested negative for serum cryoglobulins, exhibited no typical extrarenal manifestations, and achieved seroconversion of monoclonal immunoglobulins following treatment. Therefore, cryoglobulinaemia was excluded. According to the clinical diagnostic criteria for HLH-2004: fever, splenomegaly, cytopenia affecting at least two of the three blood cell lineages in the peripheral blood, hyperbilirubinemia and/or hypofibrinogenemia, increased hemophagocytosis in the bone marrow, spleen, or lymph nodes, low or absent NK cell activity, hyperferritinemia, and elevated sIL-2R levels. A total of five out of the eight criteria must be met (12). Evidence from brucellosis-associated HLH literature. A systematic review of brucellosis-associated HLH by Guo et al. (13) demonstrated that among reported cases, those with severe cytopenias, marked hyperferritinemia, and multi-organ failure often required corticosteroids with or without etoposide in addition to antibiotics. Notably, cases with rapid deterioration or neurologic involvement showed minimal response to anti-infective therapy alone and required escalation to 2004 protocol chemotherapy (13). Our patient's clinical trajectory—characterized by ferritin >2,000 ng/mL, rapidly falling platelets, and AKI requiring dialysis—aligned with this high-risk phenotype.

The patient primarily presented with a high fever unresponsive to conventional antimicrobial therapy. Imaging studies and tumor marker tests revealed no evidence of malignancy, while immunological markers, including autoantibodies, were negative, with no definitive evidence of an autoimmune disease causing the fever. Given the symptoms of fever accompanied by chills, an infectious etiology remained the primary consideration. Following screening for bacterial, viral, and fungal pathogens, the patient received a definitive diagnosis of brucellosis. The concomitant acute kidney injury presented multiple potential etiologies. Relevant case reports of Brucella infection had documented renal complications, including polycystic kidney infection, transplanted kidney infection, glomerulonephritis, renal abscess, acute renal failure, and minimal change disease nephrotic syndrome (Table 2).

**Table 2:** A literature review of case reports on brucellosis complicated by renal diseases.

Case	Gender	Age	Symptoms	Brucella species	Outcome	References
Polycystic kidney disease infection	Male	40	A 1-week history of right-sided flank pain, haematuria and fever.	Not identified	Oral doxycycline and rifampicin were administered, and following completion of the 6-week course, the polycystic kidney infection resolved.	(14)
Kidney transplant infection	Male	63	Fever and burning dysuria.	Not identified	Following successful completion of treatment, his repeat Brucella titer and blood culture results were negative.	(15)
Glomerulonephritis	Male	58	Swelling in the hands and face, itchy eyes and headache persist-ing for the previous 5 days.	Not identified	Treatment was discontinued after 6 weeks, with the patient making a completely recovery.	(16)
Renal abscess	Male	45	Fever, mild headache, myalgia in the lower limbs and back, sore gums, sweating, and an occasional cough.	Not identified	Following 20 months of treatment, blood counts, urine tests, liver and kidney function tests, and Brucella agglutination tests were all normal.	(17)
Acute renal failure	Female	45	Joint pain, fatigue, malaise, high fever for 1 week.	*Brucella melitensis*	Following 6 weeks of treatment, both biochemical and hematological parameters remained within normal ranges.	(18)
Minimal change nephrotic syndrome	Male	53	Fever, haematuria and proteinuria.	Not identified	Following 1 year of treatment, the patient achieved complete remission with no signs of recurrence of brucellosis.	(19)

Brucellosis is primarily transmitted through contact with blood, bodily fluids, and aerosols *via* the digestive tract, skin, mucous membranes, and respiratory tract, and may also be spread through consumption of unpasteurised dairy products (1, 2). In endemic regions, the hematocrit test, tube agglutination test, and blood culture should be routine investigations for patients presenting with unexplained fever to facilitate aetiological diagnosis. The acute phase of brucellosis may present with fever, arthralgia, hepatosplenomegaly, and other symptoms. The most common urogenital manifestation is epididymo-orchitis, while renal involvement is less frequent (5). Renal damage associated with Brucella infection manifests in diverse forms, including glomerulonephritis, interstitial nephritis, renal abscesses, acute kidney injury, and IgA nephropathy, all of which have been documented (20–24). Clinical manifestations predominantly include haematuria, proteinuria, and renal insufficiency, with proteinuria typically resolving following acute infection. Literature reports indicate that proteinuria resolved in all followed-up patients except those with brucellar endocarditis (25). Interstitial nephritis predominantly occurs during the acute infection phase, arising from direct bacterial invasion or treatment with anti-brucellosis medications. It typically manifests as haematuria, leukocyturia, and minimal proteinuria. Such patients respond rapidly and favorably to anti-brucellosis therapy, with a generally favorable prognosis (26). Additional studies have documented renal involvement concurrent with brucellar endocarditis, which is attributed to acute-phase circulating immune complex deposition (27).

A major limitation of this case report is the absence of renal biopsy. At the time of presentation, the patient was critically ill with severe pancytopenia, active fever, and coagulopathy, which posed a prohibitively high bleeding risk for percutaneous renal biopsy. Furthermore, the rapid clinical deterioration necessitated immediate initiation of continuous renal replacement therapy (CRRT) and combined antimicrobial therapy. Given the prompt normalization of urine protein and steady decline in serum creatinine following anti-brucellosis treatment, the clinical decision was made to forgo biopsy in favor of urgent therapeutic intervention. While this approach was clinically justified, we acknowledge that histopathological confirmation would have provided definitive evidence regarding the etiology of acute kidney injury.

The probable histopathological patterns in this patient can be inferred based on clinical, laboratory, and epidemiological data. Two primary mechanisms should be considered: First, acute tubulointerstitial nephritis (ATIN) directly induced by Brucella infection. This represents the most common renal histopathological finding in acute brucellosis, characterized by interstitial inflammatory infiltration, tubular damage, and variable proteinuria. Ceylan et al. (5) reported that among 15 patients with brucellosis-associated renal involvement, 6 had clinically diagnosed tubulointerstitial nephritis, and renal biopsy in selected cases confirmed interstitial inflammation with or without granuloma formation. Our patient presented with acute onset fever, heavy proteinuria (12 g/24 h), pyuria, and elevated serum creatinine—features consistent with ATIN. The rapid resolution of proteinuria and improvement in renal function following antimicrobial therapy further support an infectious-inflammatory etiology rather than irreversible glomerular sclerosis. Second, HLH-related renal injury secondary to systemic cytokine storm. Secondary hemophagocytic lymphohistiocytosis (HLH) triggered by brucellosis is characterized by excessive secretion of pro-inflammatory cytokines including IL-6, IFN-γ, and TNF-α, which can induce vascular endothelial damage and multi-organ dysfunction (28). In this context, renal injury may result from (1) direct cytokine-mediated tubular and glomerular endothelial injury, (2) microvascular thrombosis secondary to hyperinflammation, and (3) ischemic acute tubular necrosis due to systemic hypotension and capillary leak. The markedly elevated ferritin, hypertriglyceridemia, and profoundly elevated sCD25 in our patient indicate robust macrophage activation and T-cell dysregulation, which likely contributed to renal parenchymal damage. Notably, the coexistence of acute kidney injury and HLH in brucellosis is exceedingly rare, and the relative contribution of each mechanism to renal damage remains speculative without histopathological confirmation.

Treatment for brucella-associated renal damage primarily involves pathogenetic therapy, emphasizing early initiation, combination therapy, adequate dosage, and a full course of treatment. The currently recommended first-line regimen comprises doxycycline combined with rifampicin; if rifampicin use is contraindicated, sulfamethoxazole may be substituted. In cases with complications, triple or quadruple therapy is advised, with a favorable prognosis (29). Kadir et al. (5) through retrospective analysis, identified 15 patients with brucellosis complicated by renal impairment between 1998 and 2006 (5). Fourteen cases presented with renal failure, primarily due to tubulointerstitial nephritis. Following anti-brucella therapy, renal function fully recovered in 11 patients, demonstrating favorable outcomes (30).

A distinctive feature of this case lies in the combination of acute kidney injury with haemophagocytic syndrome, also termed haemophagocytic lymphohistiocytosis. First described by Risdall in 1979, this is a fatal hyperinflammatory syndrome commonly characterized by fever, pancytopenia, and hepatosplenomegaly—features also shared with brucellosis, rendering differential diagnosis particularly crucial (28). This condition frequently arises secondary to tumors, infections, immune disorders, organ transplantation, or CAR-T therapy, and is potentially linked to excessive cytokine secretion [including interleukin-6 (IL-6), IL-12, IL-10, and interferon-γ (IFN-γ)] triggered by infection (31). Administration of anti-Brucella antibiotics may reverse disease progression without requiring conventional chemotherapy (32). In this case, due to the rapid progression of the disease, etoposide chemotherapy was administered. Following the onset of bone marrow suppression, the drug was discontinued. With the initiation of anti-infective therapy, the haemophagocytic syndrome was effectively controlled and improved. The treatment was successful; however, a limitation was the absence of a renal biopsy and corresponding pathological data. This outcome was attributed to the patient's renal response to the treatment, as evidenced by the normalization of urinary protein levels and the continued reduction in serum creatinine concentrations.

## Conclusion

Brucellosis continues to pose a significant public health challenge in China. This case underscores the diagnostic complexity of brucellosis presenting with uncommon complications, specifically acute kidney injury and pancytopenia, which are frequently overlooked in routine clinical practice. The diagnostic process primarily depends on clinical presentation and epidemiological data; therefore, cases of unexplained fever with concurrent renal dysfunction and cytopenia necessitate heightened suspicion and systematic screening for Brucella infection to avert missed diagnoses. The acute kidney injury observed in this patient represented a rare renal manifestation of brucellosis, distinct from the more commonly reported epididymo-orchitis. While antimicrobial therapy for brucella-related renal involvement has been shown to produce favorable outcomes, vigilant monitoring for adverse reactions remains crucial. Additionally, the progressive pancytopenia in this case was attributed to secondary haemophagocytic syndrome triggered by brucellosis; its clinical manifestations were non-specific and required careful differentiation from primary hematological disorders. Management of secondary haemophagocytic syndrome necessitated targeting the underlying brucellosis infection. The coexistence of these two rare complications—acute kidney injury and pancytopenia secondary to haemophagocytic syndrome—in a single patient highlights the systemic nature of brucellosis and the imperative for comprehensive evaluation in atypical presentations. Misdiagnosis and missed diagnoses remain the most prevalent fatal factors, particularly when atypical manifestations dominate the clinical picture. At present, there is a paucity of long-term follow-up studies specifically addressing renal and hematological outcomes in brucellosis patients, highlighting the need for further research in this area.

## Data Availability

The original contributions presented in the study are included in the article/supplementary material, further inquiries can be directed to the corresponding author.
